# Solid Fuel Use and Risks of Respiratory Diseases. A Cohort Study of 280,000 Chinese Never-Smokers

**DOI:** 10.1164/rccm.201803-0432OC

**Published:** 2019-02-01

**Authors:** Ka Hung Chan, Om P. Kurmi, Derrick A. Bennett, Ling Yang, Yiping Chen, Yunlong Tan, Pei Pei, Xunfu Zhong, Jianxin Chen, Jun Zhang, Haidong Kan, Richard Peto, Kin Bong Hubert Lam

**Affiliations:** ^1^ Clinical Trial Service Unit and Epidemiological Studies Unit, Nuffield Department of Population Health, University of Oxford, Oxford, United Kingdom; ^2^ Medical Research Council Population Health Research Unit, University of Oxford, Oxford, United Kingdom; ^3^ Chinese Academy of Medical Sciences, Beijing, China; ^4^ Pengzhou Center for Disease Control and Prevention, Pengzhou, China; ^5^ Pengzhou People’s Hospital, Pengzhou, China; ^6^ Suzhou Center for Disease Control and Prevention, Suzhou, China; and; ^7^ School of Public Health, Fudan University, Shanghai, China

**Keywords:** solid fuels, chronic obstructive pulmonary disease, acute lower respiratory infection, household air pollution

## Abstract

**Rationale:** Little evidence from large-scale cohort studies exists about the relationship of solid fuel use with hospitalization and mortality from major respiratory diseases.

**Objectives:** To examine the associations of solid fuel use and risks of acute and chronic respiratory diseases.

**Methods:** A cohort study of 277,838 Chinese never-smokers with no prior major chronic diseases at baseline. During 9 years of follow-up, 19,823 first hospitalization episodes or deaths from major respiratory diseases, including 10,553 chronic lower respiratory disease (CLRD), 4,398 chronic obstructive pulmonary disease (COPD), and 7,324 acute lower respiratory infection (ALRI), were recorded. Cox regression yielded adjusted hazard ratios (HRs) for disease risks associated with self-reported primary cooking fuel use.

**Measurements and Main Results:** Overall, 91% of participants reported regular cooking, with 52% using solid fuels. Compared with clean fuel users, solid fuel users had an adjusted HR of 1.36 (95% confidence interval, 1.32–1.40) for major respiratory diseases, whereas those who switched from solid to clean fuels had a weaker HR (1.14, 1.10–1.17). The HRs were higher in wood (1.37, 1.33–1.41) than coal users (1.22, 1.15–1.29) and in those with prolonged use (≥40 yr, 1.54, 1.48–1.60; <20 yr, 1.32, 1.26–1.39), but lower among those who used ventilated than nonventilated cookstoves (1.22, 1.19–1.25 vs. 1.29, 1.24–1.35). For CLRD, COPD, and ALRI, the HRs associated with solid fuel use were 1.47 (1.41–1.52), 1.10 (1.03–1.18), and 1.16 (1.09–1.23), respectively.

**Conclusions:** Among Chinese adults, solid fuel use for cooking was associated with higher risks of major respiratory disease admissions and death, and switching to clean fuels or use of ventilated cookstoves had lower risk than not switching.

**At a Glance Commentary**

**Scientific Knowledge on the Subject**

Previous studies have suggested an association between household air pollution from solid fuel use and excess chronic obstructive pulmonary disease (COPD) risk, but the magnitude of the association varied greatly across different studies, and the evidence on respiratory diseases other than COPD in adults has been limited. Whether switching from solid to clean fuels or use of ventilation in adults may have any impact on respiratory hospitalization risk has not been examined in large-scale population-based cohort studies.

**What This Study Adds to the Field**

In this cohort study of 280,000 never-smoking Chinese adults, long-term solid fuel use for cooking was associated with significant excess risks of hospitalization and death from both acute and chronic respiratory diseases, including chronic lower respiratory disease and acute lower respiratory tract infection. The excess risk was greater among persistent wood than coal users, but smaller among those who switched from solid to clean fuels or used ventilated cookstoves. An association between solid fuel use and COPD admissions and death was found, but it was far weaker than estimates from meta-analysis of cross-sectional studies for airflow obstruction. This study also provides suggestive evidence that improved ventilation or switching to clean fuels may alleviate the excess respiratory risks associated with solid fuel use.

Household air pollution (HAP), arising mainly from domestic burning of solid fuels (e.g., coal and biomass) for cooking, is a leading cause of premature death and disease burden worldwide (1). Currently, >2.7 billion individuals, mainly those from rural areas in low- and middle-income countries (LMICs), are regularly exposed to high levels of HAP (2).

The biological plausibility (due to its resemblance to smoking) that solid fuel use is associated with higher risk of chronic obstructive pulmonary disease (COPD) in adults does not have a strong evidence base, as conclusions drawn from previous meta-analyses of studies with relatively small sample sizes were limited by high levels of heterogeneity and publication bias (3–6). In contrast, three out of the four more recent, larger studies have found no evidence of a significant association with airflow obstruction (7–10). There has also been little reliable evidence on the relationship between HAP and hospitalization or death from COPD, which is relevant to the understanding of the public health burden in LMICs such as China, where COPD is often diagnosed based on symptoms (chronic bronchitis) or radiological evidence (emphysema) rather than airflow obstruction, as spirometry is not routinely performed (8, 11). Few studies have investigated the effects of HAP on respiratory diseases other than COPD such as acute lower respiratory infection (ALRI) in adults (12, 13). We report findings on the use of solid fuels for cooking and its association with hospitalization and death from acute and chronic respiratory diseases in ∼280,000 never-smoking Chinese adults from the China Kadoorie Biobank (CKB) study.

## Methods

### Study Design

Detailed methods of the CKB study have been described previously (14–16). Between 2004 and 2008, 512,000 adults aged 30–79 years were recruited from 10 areas across China (see Figure E1 in the online supplement) and undertook a computer-assisted interview and physical measurements (including spirometry) by trained health workers following standardized procedures (14, 15). The laptop-based questionnaire incorporated stringent logic and error checks to avoid coding errors, and the quality of data collection was closely monitored, with regular feedback and further training provided to health workers (14, 15). Spirometry was performed according to the American Thoracic Society guidelines as described previously (10), but no bronchodilator was administered. Approval was obtained from the Ethical Review Committee of the Chinese Center for Disease Control and Prevention (Beijing, China) and the Oxford Tropical Research Ethics Committee, University of Oxford (Oxford, United Kingdom). Written informed consent was obtained from all participants.

### Assessment of Solid Fuel Use

At baseline, each participant was asked to recall, for up to their three most recent residences, how many years they had lived there, cooking frequency (no cooking facility/never/rarely, monthly, weekly, or daily), and ownership of ventilated cookstoves (17). Participants who cooked at least monthly, in each of their respective residences, were asked about the primary fuel type used (electricity, gas, coal, wood, charcoal, or other unspecified). If two or more fuel types were used at a residence, the one used most frequently and for the longest duration was recorded. Clean fuels included electricity or gas, whereas solid fuels comprised coal or wood (including charcoal because of their compositional and emission similarities) (12). Participants cooking weekly or daily were considered as cooking regularly (90% of whom cooked daily at baseline), and their HAP exposure at each residence was classified according to the primary fuel type. Long-term exposure was assessed by grouping participants who used the same primary fuel type throughout their three residences and those who had switched from solid to clean fuels before baseline separately. Long-term solid fuel users were further categorized into three groups (always coal, always wood, and a mixture of coal and wood), along with the estimated duration of continuous exposure to solid fuels for cooking during the recall period (<20, 20–39, and ≥40 yr). To explore the potential impact of ventilated cookstove use, a three-category composite exposure was derived (clean fuels, solid fuels with ventilated cookstoves, and solid fuels without ventilated cookstoves). Further details on exposure assessment are available online (Supplementary Methods section E1).

### Follow-up for Mortality and Morbidity

All participants were followed up through electronic linkage, using unique personal identification numbers, to established death and morbidity registries and to a nationwide health insurance system (∼99% coverage in the study areas), which provided coded fatal and nonfatal events (International Classification of Diseases, 10th revision [ICD-10]) (15). The endpoints investigated in this study include the first hospitalization event (during the follow-up period) or death from major respiratory diseases (including chronic lower respiratory disease [CLRD; ICD-10 J40–J47, where J41–J44 were considered as COPD], acute lower respiratory infection [ALRI; J12–J18 and J20–J22], acute upper respiratory infection [AURI; J00–J06], and other upper respiratory disease [J30–J39]) and death from any respiratory diseases (excluding those due to external agents: J00–J47, J80–J94, J96–J99). Participants without the above events were censored upon death, loss to follow-up, or January 1, 2016. To verify the validity of COPD diagnoses, a random sample of ∼1,000 COPD cases (∼10%) between 2004 and 2013 was adjudicated by respiratory physicians independently (18). Only 14% of the COPD cases had pre–bronchodilator spirometry performed. However, most (85%) COPD diagnoses were considered to be adequately supported by different sources of evidence based on clinical symptoms, risk exposure, radiological examinations, or spirometry in accordance with the existing clinical guidelines (18).

### Statistical Analysis

Our analyses were restricted to never-smokers (*n* = 317,614), defined as those who had either never smoked or had smoked <100 cigarettes or equivalent during their lifetime. We excluded participants with unreliable recall information on residence duration (*n* = 1,573) and those with self-reported doctor-diagnosed major chronic diseases (chronic bronchitis, emphysema, tuberculosis, asthma, any cancer, stroke, transient ischemic attack, or coronary heart disease) prior to the baseline survey (*n* = 26,095). Participants who used other unspecified fuels at any residence (*n* = 2,527), those who switched from clean to solid fuels (*n* = 655), or those who had cooked previously but stopped at baseline (*n* = 8,926) were also excluded, leaving 277,838 participants in the final study population.

Direct standardization yielded age-, sex-, and study area–adjusted percentages or means of baseline characteristics for long-term cooking fuel exposure categories. We used Cox regression to estimate hazard ratios (HRs) and 95% confidence intervals (CIs) for first hospitalization or death from respiratory disease in association with long-term solid fuel use for cooking (referred to as risk of respiratory disease in the subsequent text), stratifying for age-at-risk (5-yr intervals), sex, and study area (10 areas), and adjusted for education (no formal school, primary school, middle school, or high school/college/university), household income (<10,000, 10,000–19,999, 20,000–34,999, or ≥35,000 yuan), occupation (agricultural worker, factory worker, non-manual worker, or others), alcohol consumption (never/rarely, occasional, ex-drinker or reduced intake, or weekly regular), body mass index (BMI; continuous), environmental tobacco smoke (ETS) exposure (<1 d/wk, 1–5 d/wk, or daily or almost every day), cookstove ventilation (all stoves, some stoves, or none), primary heating fuel exposure (always clean fuels, solid to clean fuel, always solid fuels, or others), and length of recall period (continuous), where appropriate. Fuller details of the selection process used for confounders for adjustment are provided online (Supplementary Methods section E2). The proportional hazard assumption was confirmed to be upheld by using standard methods (19). For exposure measures with more than two categories, a group-specific CI of the HR was calculated from the variance of the log hazard in each category (including the reference category) as described previously (16, 20), and more details are provided online (Supplementary Methods section E3). The cumulative probability of being hospitalized or dying from each specific cause during follow-up is presented using Kaplan-Meier plots.

We conducted subgroup analyses by baseline characteristics (birth year, age, sex, education, ETS, alcohol consumption, BMI, leg length, years of having a refrigerator at home [the latter two are proxies for the early life environment]). We performed further sensitivity analyses to reduce the potential impact of reverse causation and residual confounding by excluding *1*) participants with <20 years of recall period (“frequent movers,” *n* = 26,742), *2*) participants with poor self-reported health at baseline (*n* = 26,551), *3*) participants who cooked weekly at baseline (*n* = 25,466), and *4*) individuals with spirometry-defined airflow obstruction (*n* = 15,879) or chronic respiratory symptoms (*n* = 4,842) at baseline, respectively. Details of the assessment and definitions of airflow obstruction and chronic respiratory symptoms are available online (Supplementary Methods section E4). All analyses were conducted using SAS software version 9.3.

## Results

Among the 277,838 never-smoking participants, the mean (SD) age was 50.3 (10.3) years and 91% were female. The mean total duration of the three most recent residences was 39.7 (14.5) years, with 91% participants having had at least 20 years of residence covered. Among 91% who reported regular cooking during the recall period, 52% used solid fuel throughout. Compared with long-term clean fuel users, solid fuel users were older, less educated, had lower income, were more likely to live in rural areas and to report poor general health status, and were less likely to use ventilated cookstoves. There was no major difference in BMI or exposure to ETS between the two groups (Table 1).

**Table 1.** Baseline Characteristics of Never-Smoking Participants by Long-Term Primary Cooking Fuel Exposure

Characteristic	Always Clean	Solid to Clean	Always Solid	Never Cooked Regularly	All Participants
*n*	53,130	66,115	131,270	27,323	277,838
Age, yr, mean (SD)	45.3 (9.5)	50.9 (9.8)	53.0 (10.2)	45.6 (11.2)	50.3 (10.3)
Female sex, %	86.8	97.0	95.5	40.7	90.9
Urban residence, %	88.0	79.2	8.5	49.8	44.3
No formal education, %	14.5	18.8	28.7	20.0	23.6
Household income <10,000 yuan/yr, %	18.3	20.4	37.8	22.6	28.6
Occupation, %					
Agricultural worker	19.7	26.6	48.4	31.0	41.3
Factory worker	13.1	12.1	11.1	15.9	12.0
Non–manual worker	17.9	13.9	6.6	16.2	9.9
Others*	49.3	47.4	34.0	36.8	36.9
Current drinker in males, %	21.3	21.5	18.5	19.6	19.1
Current drinker in females, %	2.0	1.7	1.5	2.0	1.6
Environmental tobacco smoke, %					
<1 d/wk	44.9	39.6	39.4	41.9	40.5
1–5 d/wk	17.8	19.1	18.8	17.3	19.0
Daily or almost every day	37.3	41.4	41.8	40.8	40.4
Cookstove ventilation, %					
All stoves	61.1	55.8	22.8	47.9	44.7
Some stoves	19.7	24.4	46.5	28.3	31.9
None	19.2	19.9	30.7	23.8	23.5
Body mass index, kg/m^2^, mean (SD)	23.8 (3.3)	24.2 (3.4)	23.6 (3.4)	23.7 (3.2)	23.8 (3.4)
Systolic blood pressure, mm Hg, mean (SD)	127.9 (19.9)	128.7 (21.4)	130.2 (22.2)	128.4 (20.3)	129.7 (21.6)
Self-reported poor health, %	8.3	8.2	10.4	9.7	9.1

Means and percentages were adjusted for age, sex, and study area when appropriate. Participants who switched from clean to solid fuels, used unspecified fuels, or cooked regularly but stopped were excluded from analysis (*n* = 12,108).

* “Others” in occupation include housewife/husband, retired, self-employed, unemployed, or other unspecified.

**Figure 1.**
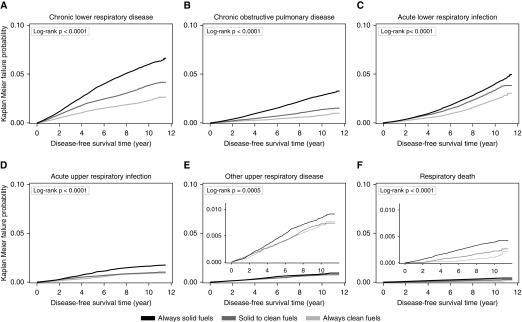
Kaplan-Meier probabilities of developing specific respiratory diseases during follow-up.

During 2.6 million person-years of follow-up (mean, 9.1 [1.4] yr), 19,823 first hospitalization events and deaths from major respiratory diseases were recorded, including 10,553 CLRD, 4,398 COPD, 7,324 ALRI, and 3,011 AURI. Figure 1 presents the Kaplan-Meier probability of hospitalization or death from each cause-specific outcome across the three main exposure categories (always clean, solid to clean, or always solid). Compared with long-term clean fuel use, long-term solid fuel use for cooking was associated with higher risks of several major respiratory diseases, with adjusted HRs of 1.36 (group-specific 95% CI, 1.32–1.40) for all major respiratory diseases, 1.47 (1.41–1.52) for CLRD, 1.10 (1.03–1.18) for COPD, 1.16 (1.09–1.23) for ALRI, 1.59 (1.48–1.71) for AURI, 1.56 (1.40–1.73) for other upper respiratory disease, and 1.56 (1.28–1.89) for respiratory death. The HRs were significantly weaker in participants who switched from solid to clean fuels than those who used solid fuels persistently (for major respiratory disease, 1.14 [1.10–1.17] vs. 1.36 [1.32–1.40]) (Table 2). For major respiratory diseases, the corresponding HR was similar in men and women (1.46 [1.30–1.63] vs. 1.37 [1.32–1.41]) and across a range of baseline characteristics (Table E1).

**Table 2.** Incidence Rates and Adjusted Hazard Ratios for Hospitalization or Death from Major Respiratory Diseases by Long-Term Cooking Fuel Exposure

	Number of Events	Rate (*per 100,000 Person-Years*)*	HR (95% CI)^†^
Major respiratory diseases^‡^			
Always clean	2,576	797	1.00 (0.96–1.04)
Solid to clean	4,575	891	1.14 (1.10–1.17)
Always solid	12,672	1,088	1.36 (1.32–1.40)
Chronic lower respiratory disease^§^			
Always clean	1,093	371	1.00 (0.94–1.07)
Solid to clean	2,271	444	1.20 (1.15–1.26)
Always solid	7,189	619	1.47 (1.41–1.52)
Chronic obstructive pulmonary disease^||^			
Always clean	357	192	1.00 (0.89–1.12)
Solid to clean	778	167	0.96 (0.89–1.03)
Always solid	3,263	222	1.10 (1.03–1.18)
Acute lower respiratory infection^¶^			
Always clean	1,037	344	1.00 (0.93–1.07)
Solid to clean	1,871	308	1.08 (1.02–1.13)
Always solid	4,416	328	1.16 (1.09–1.23)
Acute upper respiratory infection**			
Always clean	444	108	1.00 (0.90–1.11)
Solid to clean	584	149	1.13 (1.04–1.23)
Always solid	1,983	194	1.59 (1.48–1.71)
Other upper respiratory disease^††^			
Always clean	327	75	1.00 (0.89–1.13)
Solid to clean	424	70	1.10 (0.99–1.22)
Always solid	984	113	1.56 (1.40–1.73)
Respiratory death^‡‡^			
Always clean	51	17	1.00 (0.75–1.33)
Solid to clean	126	14	0.96 (0.78–1.19)
Always solid	457	38	1.56 (1.28–1.89)

*Definition of abbreviations*: CI = confidence interval; HR = hazard ratio; ICD-10 = International Classification of Diseases, 10th revision.

* Event rates were adjusted for age, sex, and study area structure of the China Kadoorie Biobank study population.

^†^ Hazard ratios were stratified for age at risk, sex, and study area and adjusted for education, household income, occupation, alcohol consumption, body mass index, environmental tobacco smoke, cookstove ventilation, heating fuel, and length of recall period.

^‡^ ICD-10 codes J00–J06, J12–J18, J30–J22, J30–J39, and J40–J47.

^§^ ICD-10 codes J40–J47.

^||^ ICD-10 codes J41–J44.

^¶^ ICD-10 codes J12–J18 and J20–J22.

** ICD-10 codes J00–J06.

^††^ ICD-10 codes J30–J39.

^‡‡^ ICD-10 codes J00–J47, J80–J94, and J96–J99.

**Figure 2.**
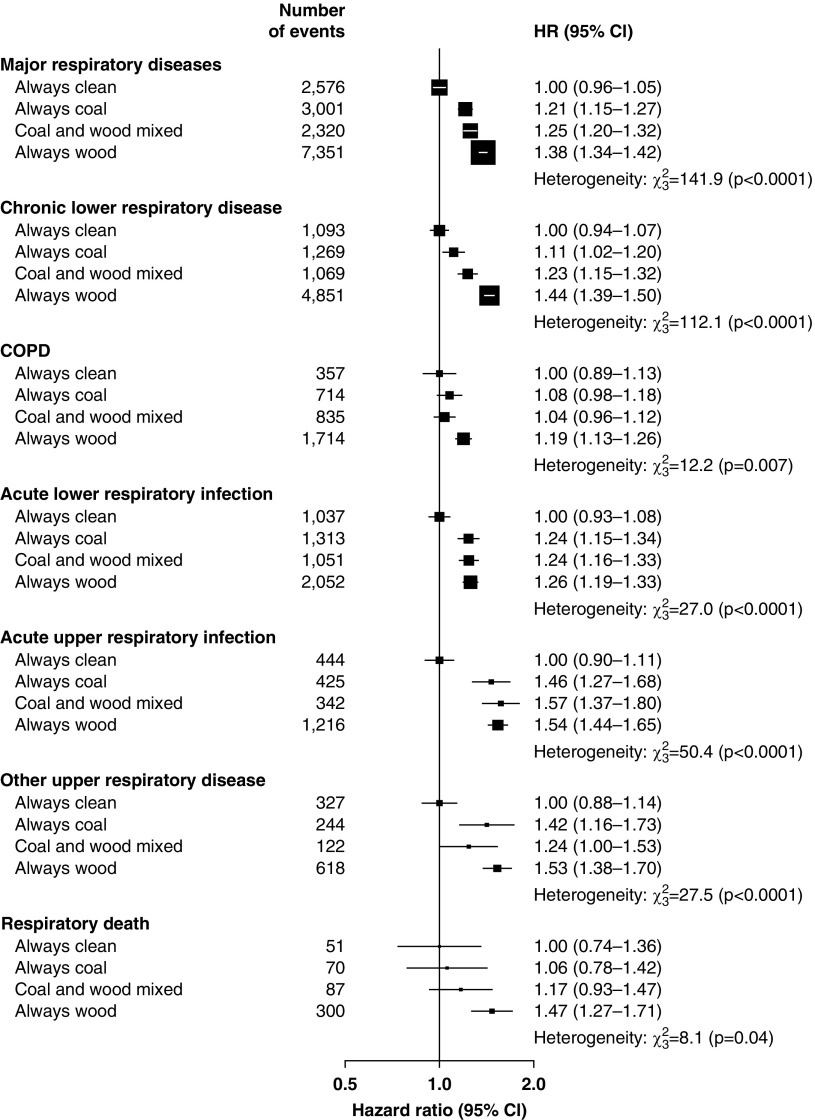
Adjusted hazard ratios for major respiratory diseases by duration of continuous exposure to solid cooking fuel in never-smokers. Hazard ratios were stratified by age at risk (in 5-yr groups), sex, and study area and were adjusted for education, household income, occupation, alcohol consumption, body mass index, environmental tobacco smoke, cookstove ventilation, primary heating fuel exposure, and length of recall period. The black boxes represent hazard ratios, with the size inversely proportional to the variance of the logarithm of the hazard ratio, and the horizontal lines represent 95% confidence intervals. CI = confidence interval; COPD = chronic obstructive pulmonary disease; HR = hazard ratio.

**Figure 3.**
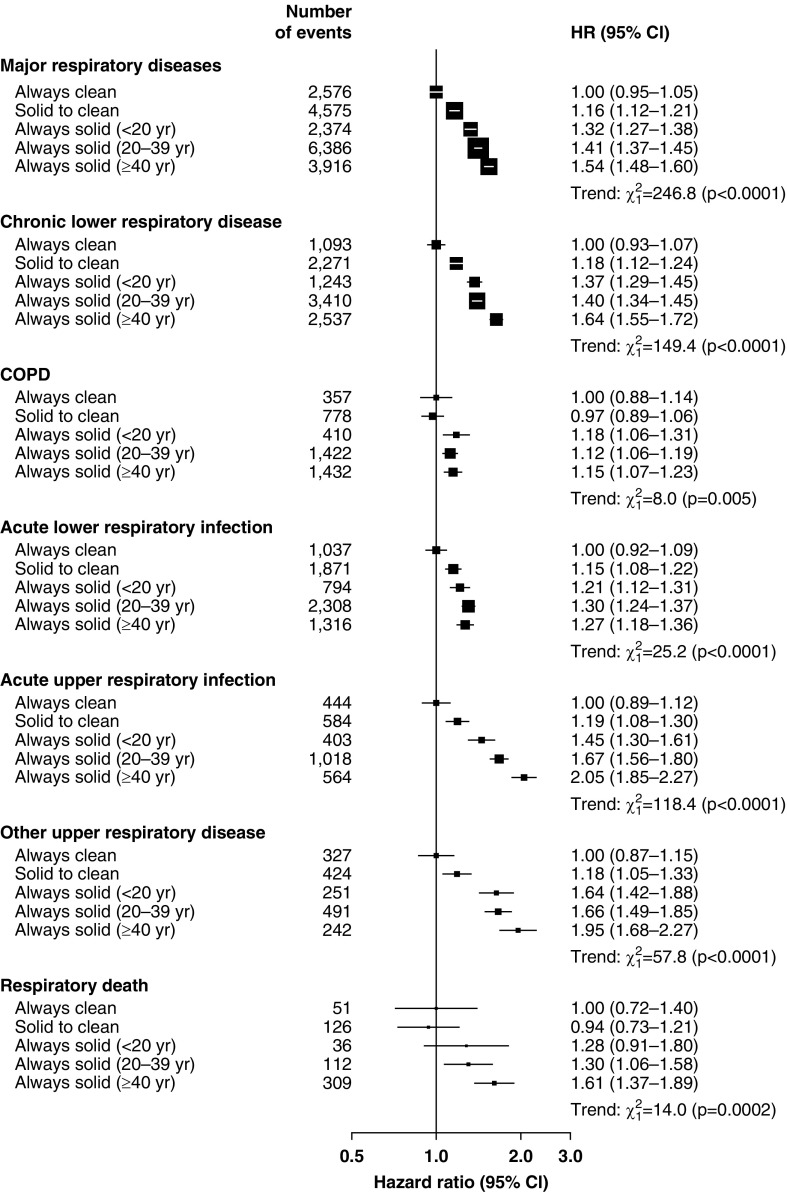
Adjusted hazard ratios for major respiratory diseases by type of primary cooking fuel used in never-smokers. Conventions are as in Figure 2. For definition of abbreviations, *see* Figure 2.

**Figure 4.**
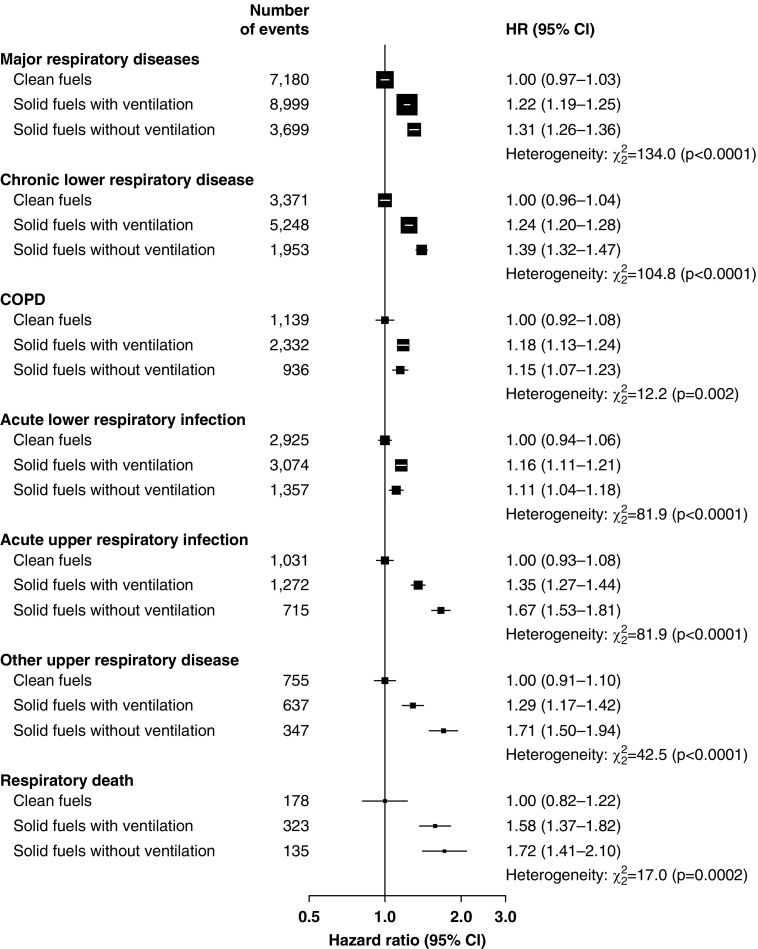
Adjusted hazard ratios of major respiratory diseases associated with primary cooking fuel and use of ventilated cookstoves at baseline. Conventions are as in Figure 2 except that the hazard ratios were not adjusted for cookstove ventilation and length of recall period. For definition of abbreviations, *see* Figure 2.

Compared with participants who had always used clean fuels for cooking, the risk of major respiratory diseases increased with duration of persistent solid fuel use, with HRs of 1.32 (1.26–1.39), 1.41 (1.37–1.45), and 1.54 (1.48–1.60) in those who used solid fuels for <20, 20–39, and ≥40 years, respectively (*P*_trend_ < 0.0001). Similar relationships were observed for each specific respiratory disease (*P*_trend_ ≤ 0.003 for all comparisons) (Figure 2). Among long-term solid fuel users for cooking, those who used wood had higher HRs for major respiratory diseases than did those who used coal (1.37 [1.33–1.41] vs. 1.22 [1.15–1.29]), and those who switched between wood and coal had an intermediate risk (1.25 [1.19–1.31]). Similar patterns of association were observed for CLRD, COPD, ALRI, and respiratory death but not for other respiratory disease outcomes (Figure 3). Excess risk of major respiratory diseases among the solid fuel users with ventilated cookstoves were significantly lower compared with those who used unventilated cookstoves (1.22 [1.19–1.25] vs. 1.29 [1.24–1.35]). Similar associations were observed for CLRD, AURI, other upper respiratory disease, and respiratory death (Figure 4).

The strength of observed associations between solid fuel use for cooking and most respiratory diseases did not change substantially after excluding frequent movers, participants with poor self-reported health, those who cooked weekly, or those who had signs of airflow obstruction or chronic respiratory symptoms at baseline (Table E2).

## Discussion

In this large study of 280,000 never-smoking Chinese adults who had no known prior history of major chronic diseases at baseline, long-term use of solid fuels for cooking was associated with significant elevated risks of hospitalization or death from both acute and chronic respiratory diseases, with consistent results in men and women and across a range of population subgroups. The excess risks appeared to be greater among those who used wood compared with coal. Switching from solid to clean fuels or use of ventilated cookstoves was associated with relatively smaller excess risks.

Most previous epidemiological studies on solid fuel use and respiratory diseases focused on COPD in adults, with most of them being cross-sectional or case-control studies examining airflow obstruction as the outcome (3–6, 9, 21, 22). Earlier pooled analyses of these studies, often with small sample sizes, reported large excess risks (summary odds ratios from 1.94 to 2.80) (3–6), but strong evidence of publication bias (*P* < 0.007) and high levels of heterogeneity (*I*^2^ = 85%) has been found (5). Four larger and more recent population-based cross-sectional studies involving 13,000 to 67,000 participants, including two conducted in China, reported much weaker associations (from no association to ∼40% excess risk) with airflow obstruction (7–9, 22). In contrast, the present study of 280,000 Chinese never-smokers found that long-term use of solid fuel for cooking was associated with ∼10% excess risk of COPD hospitalization or death. The cohort design of this study enabled us to take account of the influence of reverse causation by excluding those with a prior history of major respiratory diseases, signs of airflow obstruction, or chronic respiratory symptoms, and by examining prospectively recorded hospitalizations or deaths. Furthermore, our analyses were restricted to never-smokers, so the residual confounding from smoking, a leading cause of COPD, should be minimized.

Many previous studies on COPD, including a previous cross-sectional analysis of CKB (10), examined spirometry-defined airflow obstruction, the hallmark of COPD, as the outcome. In the present study we focused on hospitalization and death, as there has been little information on the risk of respiratory hospitalizations and deaths associated with long-term HAP. Indeed, the low utility of spirometry for diagnosing COPD in China (7–10%) (8, 23) means many asymptomatic and mild airflow obstruction cases not requiring medical attention were less likely to have been identified, diagnosed, and captured in our records as COPD. Underdiagnosis of COPD is disproportionately higher in rural China (8), where solid fuel use is more prominent. The higher likelihood of undiagnosed cases in the exposed group means that the observed risks for COPD may well be diluted. In this regard, we observed a stronger association between long-term solid fuel use for cooking and CLRD (HR, 1.47 [95% CI, 1.41–1.52]), which included all COPD cases plus mostly unspecified bronchitis (ICD-10 J40; *n* = 7,471). It is possible that many of these unspecified bronchitis cases (but not acute bronchitis as included within ALRI) could be mild, early stages COPD or acute exacerbations of preexisting, but previously undetected, COPD, given that spirometry is rarely used for diagnosis in China. Nevertheless, this may also suggest that solid fuel use is more strongly associated with chronic bronchitis (or mucus hypersecretion in general) than with emphysema or other COPD phenotypes, which has been suggested in previous studies (6, 9, 24).

For non-COPD respiratory diseases, previous evidence has been more limited. Two small cohort studies on respiratory death (with 155 cases) and ALRI (with 229 participants, no case numbers were given) reported inconclusive findings (25, 26). A recent systematic review (13) of eight relevant studies on ALRI, most of which involved <1,000 disease events, found no consistent evidence. Our study included much larger numbers of events than all previous studies combined (∼7,300 ALRI, 3,000 AURI). We found strong evidence that long-term solid fuel use is associated with significantly elevated risk of hospitalizations or deaths from ALRI and AURI in adults. This highlights the potential need of considering adult ALRI when assessing the disease burden related to HAP exposure. It is worth noting that ALRI and AURI are acute recurring conditions. The observed associations reflect an overall shorter time to the first documented infection during the follow-up in solid fuel users, which may indirectly imply a higher rate of recurrent infection among them. Future analysis focusing on recurrent events (including acute exacerbations of COPD) should be able to clarify this.

Most previous studies on COPD have examined biomass (mostly wood) only, whereas we analyzed both coal and wood (combined as “solid fuels” and separately), the latter of which has been linked to higher levels of particulate pollution and possibly higher risk of COPD (6, 12). Consistently, the risks of CLRD, COPD, and ALRI in our study were higher among those that persistently used wood compared with those using coal. However, an earlier cross-sectional analysis of CKB on the prevalence of airflow obstruction found seemingly protective effects of wood burning (OR, 0.91 [95% CI, 0.86–0.98]) and a deleterious effect of coal use (1.10 [1.02–1.20]) at baseline in women (10). The two studies differ importantly by the disease outcome examined (prevalence of spirometry-detected airflow obstruction [10] vs. rate of clinical episodes of COPD), as well as inclusion criteria, exposure classification, and analysis strategy. In the current study participants with any prior chronic diseases were excluded. We classified individuals who cooked weekly or daily as regular users of fuels (clean or solid), whereas the previous analysis included also less frequent (monthly) cooks (who were more likely to be men, factory workers, and clean fuel users compared with the more frequent cooks). Furthermore, the current study has additionally adjusted for other important confounders that were not taken into account in the previous study (e.g., ETS, occupation, BMI). For upper respiratory disease, the excess risks appeared to be broadly similar in the long-term wood and coal users for reasons that are not fully understood. It is possible that the etiology or mechanisms between chronic respiratory disease and respiratory infections in relation to air pollutants generated by burning of different fuel types may differ. Further investigations including direct measurement of HAP and characterization of smoke constituents are planned and should help to clarify our findings.

It has been reported in both observational and intervention studies that HAP exposure and acute respiratory symptoms in adults may be reduced through adequately maintained cookstove ventilation (27). However, there has been no clear evidence on the long-term respiratory benefits of improved cookstove ventilation in adults (27). A retrospective cohort study involving 42,000 Chinese adults reported significantly lower risks of pneumonia mortality (225 cases) and self-reported physician diagnosis of COPD (1,487 cases) in lifelong coal users for cooking who adopted a ventilated cookstove compared with those who did not (28, 29). In contrast, another cohort study of 600 Chinese adults (74 cases) found no significant effect of improved ventilation on the risk of airflow obstruction (30). In our study, solid fuel users who used ventilated cookstoves had lower risks of CLRD and upper respiratory diseases, but not ALRI, COPD, or respiratory death, compared with those who used unventilated cookstoves. This is in agreement with existing evidence that improved ventilation generally may have more prominent benefits on mild, acute conditions but not on more severe diseases such as COPD or ALRI, possibly because the HAP levels after improvement remain substantially above the recommended threshold (27, 31). The discrepancy in the results on CLRD and COPD, as discussed above, may be related to the unspecified bronchitis (ICD-10: J40) that could be acute exacerbation of early stages of COPD. Future large-scale randomized controlled trials with long follow-up and appropriately designed interventions are needed to assess the effect of using ventilated cookstoves on major respiratory conditions such as ALRI or COPD in adults.

Compared with the long-term persistent solid fuel users, participants who had switched their primary cooking fuel from solid to clean fuels prior to the baseline survey had smaller excess risks of all respiratory diseases studied. Although limited, there is consistent trial evidence that switching from solid to clean fuels is associated with markedly greater HAP reduction than adopting improved ventilation (32). Our findings offer supportive evidence that clean fuel adoption may be beneficial for the prevention of acute and chronic respiratory conditions. Although this might seem intuitive, it highlights that the elevated risks associated with historical solid fuel use may still be attenuated by switching to clean fuels later in life, a phenomenon similar to that of smoking cessation (16). This should encourage greater efforts to facilitate universal access to clean energy especially in LMICs, as promoted in the United Nations Sustainable Development Goal 7 (33).

The key strengths of this study lie in the large number of never-smokers, comprehensive investigation of prospectively documented hospitalization and death of a range of respiratory diseases, and the high consistency of exposure–outcome relationships across these diseases and across different population subgroups. Moreover, two common limitations of previous research on this topic, namely reverse causality and residual confounding from smoking, were carefully dealt with in this study. However, our study has several limitations that need to be taken into consideration. First, our outcome was based on linkages to hospitalization records and death certificates. Misclassification due to misdiagnosis is possible, especially for COPD owing to the low utility of spirometry in China. Although we have excluded participants with preexisting chronic diseases, admissions for COPD were unlikely to represent new onset “incident” cases, as COPD has a prolonged development period with risk factors that could trace back to preconception, meaning that it is difficult to establish temporality accurately. Nevertheless, the aim of this study was to investigate whether HAP may be associated with respiratory admissions and deaths, rather than the development of incident cases. We have also excluded those with signs of airflow obstruction at baseline or poor self-reported health in the sensitivity analyses, and the results persisted. Second, HAP exposure was estimated by self-reports of the main type of fuel used as in many other previous studies. It is possible that historical or concurrent exposure to solid fuel emission from secondary or neighborhood fuels could have elevated the background risks of clean fuel users, but we lack data on these, or from direct exposure measurement to more accurately assess exposure–response relationships. Third, instead of prospectively monitoring lifetime exposure, we were only able to estimate long-term exposure based on recall information on the three most recent residences of our participants. This might have resulted in misclassification, especially among clean fuel users who might have used solid fuels in their early life. However, the recall period covered was on average 40 years (≥70% of the adulthood in 80% of participants), and the exclusion of participants with <20 years of recall information provided gave similar findings with all participants included. Fourth, residual confounding from early-life exposure and ETS is possible owing to the lack of direct early-life exposure data and the relatively crude adjustment on ETS (based on self-reported frequency of exposure). Nonetheless, the associations observed were consistent across subgroups defined by proxies of early-life exposures (leg length, education level, years of having a refrigerator at home), and additional adjustment for duration of exposure to ETS did not alter the relationship of interest (data not shown). Finally, our study sample has an imbalanced sex ratio (9:1), and one may argue that the findings may not be generalizable to men. However, in the sex-specific analyses (with >26,000 men), we found no evidence of heterogeneity.

In conclusion, in Chinese adults, solid fuel use for cooking was associated with higher risks of admissions and death for both acute and chronic respiratory diseases, with the excess risk seemingly greater for wood than coal users, especially for CLRD, and in those with more prolonged use. A much weaker association with COPD was observed as compared with the earlier meta-analysis estimates used in global disease burden estimation. Moreover, use of ventilated cookstoves and switching to clean fuels were associated with smaller excess risks of some respiratory diseases associated with solid fuel use, reinforcing the need for strengthening the existing global initiatives to improve access to clean energy and to distribute improved cookstoves in communities where a complete switch to cleaner fuels is not yet feasible.

## Supplementary Material

Supplements

Author disclosures
