# Supplementary material for: Solid Fuel Use and Risks of Respiratory Diseases. A Cohort Study of 280,000 Chinese Never-Smokers
Source: Am J Respir Crit Care Med. 2019 Feb 1;199(3):352–61. doi: 10.1164/rccm.201803-0432OC (PMC6363974; doi:10.1164/rccm.201803-0432OC)
Supplement: Supplements [file rccm.201803-0432OC.html]

Solid Fuel Use and Risks of Respiratory Diseases. A Cohort Study of 280,000 Chinese Never-Smokers | American Journal of Respiratory and Critical Care Medicine

- disclosures.pdf (221 KB)
- chan\_data\_supplement.pdf (2 MB)
