# Supplementary material for: Solid Fuel Use and Risks of Respiratory Diseases. A Cohort Study of 280,000 Chinese Never-Smokers
Source: Am J Respir Crit Care Med. 2019 Feb 1;199(3):352–61. doi: 10.1164/rccm.201803-0432OC (PMC6363974; doi:10.1164/rccm.201803-0432OC)
Supplement: Supplements [file rccm.201803-0432OC_chan_data_supplement.pdf]

Solid Fuel Use and Risks of Respiratory Diseases: A Cohort Study of 280,000 Chinese Never-Smokers

Ka Hung Chan, Om P Kurmi, Derrick A Bennett, Ling Yang, Yiping Chen, Yunlong Tan, Pei Pei, Xunfu Zhong, Jianxin Chen, Jun Zhang, Haidong Kan, Richard Peto, Kin Bong Hubert Lam, Zhengming Chen, on behalf of the China Kadoorie Biobank Collaborative Group

Online Data Supplement

Supplementary methods

E1: Estimation of duration of exposure to solid fuel use

To assess the potential exposure-response relationship between duration of solid fuel use and risk of respiratory disease, a semi-quantitative indicator of duration of continuous exposure to solid fuels was derived. Among participants who reported primarily using solid fuels for cooking throughout the recall period (i.e. up to three most-recent residences), the duration of continuous exposure to solid fuels was calculated by summing the duration of residence (in years) in consecutive residences where solids fuels were reported as the primary fuel type for cooking. For example, if participant X lived in the three most-recent residences for 10, 15, and 25 years and cooked regularly primarily using solid fuels (coal or wood) throughout the three residences, the duration of continuous exposure to solid fuels for cooking would be 10+15+25 = 50 years. In contrast, if X reported cooking regularly using solid fuels in the two most recent residences but *not* the earliest one, the duration of continuous exposure would be 15+25 = 40 years.

For prospective analyses, the long-term solid fuel users were categorized into three groups based on their estimated duration of continuous exposure to solid fuels for cooking (<20 years, 20-39 years, ≥40 years), and their risks of respiratory disease incidence and deaths were compared with participants who primarily used solid fuels for cooking in an earlier residence but adopted clean fuels in the more recent one(s) and those who have used primarily clean fuels throughout the three residences (reference category). The residential duration among the latter two categories of participants were not considered because their exposure profile should be distinct from the long-term solid fuel users, with second lowest exposure in the fuel switchers and the lowest exposure in the reference group. Nonetheless, sensitivity analysis was conducted to exclude participants with a short recall period (<20 years) to assess the risk of bias related to the varying length of recall period between participants.

Note that in our analysis we have excluded a small number of participants who used other unspecified fuels for cooking at any residence (n = 2,527), those who reported inconsistent fuel types in the three residences (e.g. switching between clean and solid fuels; n = 655), or cooked regularly in an earlier residence but stopped in the baseline residence (n = 8,926).

E2: Modelling strategy of the main analyses

For the main analyses presented in this study, we used Cox regression to estimate hazard ratios (HRs) and 95% confidence intervals (CIs) for respiratory disease in association with long-term solid fuel use for cooking, stratifying for age-at-risk (five-year intervals), sex, and study area, and adjusted for education, household income, occupation, alcohol consumption, body mass index (BMI), environmental tobacco smoke (ETS), cookstove ventilation, primary heating fuel exposure, and length of recall period, where appropriate.

The use of age-at-risk, sex-, and study area-stratified Cox models is a standard method used in CKB. Stratification on age-at-risk is an established method to thoroughly control for the confounding effects from both age and birth cohort, and the time-dependency of the association between age and outcome of interest.(1) Stratifications of study area and sex were needed to account for the inter-region and between-sex variability of both measured and unmeasured potential confounders related to the two characteristics.

We have identified a range of other potential confounders based on the current literature and they were assessed for their contribution to the model fit, using likelihood ratio test comparing the model with and without each covariate. In particular, length of recall period was considered as an important potential confounder needs to be adjusted regardless of whether it contributed to the model fit or altered the association of interest. The largest set of significant covariates was included in the final models for all outcomes studied. Adjustment for diet, physical activity levels, leg length, self-reported health status did not significantly alter the associations of interest and were therefore not included in the final model of the manuscript.

E3: Calculation of group-specific confidence intervals

For exposure measures with more than two categories, group-specific CIs of HRs were calculated from the variance of the log hazard in each category (including the reference category) of the exposure variable. The group-specific CIs enable comparisons of HR across any two categories of exposure instead of just between a fixed reference group and other exposure categories. To calculate a conventional confidence interval comparing any two categories, with a group-specific HR (95% CI) of 1.00 (a to b) for the reference group and x (y to z) for the comparison group, the conventional CI for the comparison group would be ($\sqrt{[xy/k]}$ to $\sqrt{[xyk]}$), where $\log(k) = \sqrt{(\log2[y/x] + \log2[b/a])}$.(2)

E4: Assessment and definitions of airflow obstruction and chronic bronchitis at baseline

At baseline, two forced expiratory maneuvers using a handheld Micro Spirometer (MS01; CareFusion UK, Basingstoke, UK) were performed without the use of bronchodilator by trained technicians. The highest values of forced expiratory volume in 1 s (FEV_1) and forced vital capacity (FVC), not necessarily from the same maneuver, were used in the analyses. Further details have been described previously.(3) We defined possible airflow obstruction at baseline as FEV_1:FVC ratio < lower limit of normal estimated using the Global Lung Initiative reference equation for east-Asian.(3)

Also at baseline participants reported the presence of chronic respiratory symptoms (cough with phlegm for over three months in the past 12 months).(4) This was independent of the self-reported history of physician-diagnosed chronic bronchitis and/or emphysema.

Table E1. Adjusted HRs for major respiratory diseases by long-term primary cooking fuel exposure (always solid versus clean fuels), according to selected baseline characteristics

	Number of events				
	**Always solid fuels**	**Always clean fuels**	**HR (95% CI)***	**χ^2†**	**P-value†**
**Birth year**					
Before 1952	5,775	786	1.44 (1.32-1.58)		
1952-1961	4,203	907	1.27 (1.15-1.39)		
After 1961	2,694	883	1.39 (1.25-1.54)	0.5	0.47
**Age (years)**					
< 50	1,277	4,207	1.37 (1.26-1.50)		
50-59	4,530	765	1.23 (1.11-1.35)		
≥ 60	3,935	534	1.53 (1.37-1.71)	1.4	0.24
**Sex**					
Men	360	240	1.44 (1.20-1.74)		
Women	12,312	2,336	1.36 (1.29-1.45)	0.3	0.57
**Education**					
No formal	5,382	494	1.25 (1.13-1.38)		
Primary school	5,041	578	1.48 (1.34-1.64)		
Middle school or above	2,249	1,504	1.40 (1.26-1.56)	2.5	0.12
**Environmental tobacco smoke**					
< 1 day/ week	1,336	5,130	1.37 (1.27-1.47)		
1-5 days/ week	2,381	476	1.41 (1.24-1.59)		
Daily or almost everyday	5,161	764	1.25 (1.12-1.39)	1.4	0.23
**Leg length (cm)**					
<69.7	4,685	626	1.34 (1.20-1.49)		
69.7-72.9	4,290	838	1.42 (1.29-1.56)		
≥ 73	3,697	1,112	1.34 (1.23-1.46)	<0.1	0.96
**Years of having a refrigerator at home**					
No Fridge (0 year)	9,231	606	1.31 (1.20-1.44)		
1-9	2,834	552	1.18 (1.06-1.32)		
≥ 10	607	1,418	1.20 (1.05-1.38)	1.7	0.20
**Overall**	12,672	2,576	1.36 (1.29-1.44)		

* Hazard ratios were stratified for age-at-risk, sex and study area and adjusted for education, household income, occupation, alcohol consumption, body-mass index, environmental tobacco smoke, cookstove ventilation, heating fuel, and length of recall period, where appropriate. Conventional CIs which take into account of the variance of the reference category (always clean fuels) in each subgroup are presented to allow cross-subgroup comparison.
† χ^2 and p-values of test for trend (for subgroups of three categories) and heterogeneity (for subgroups of two categories)

Table E2. Adjusted hazard ratios (HRs) for respiratory events by long-term primary cooking fuel exposure - sensitivity analyses

Events	(1) Excluded frequent movers (<20 years recall)		(2) Excluding poor self-reported health		(3) Excluding weekly-regular cooks*		(4) Excluding baseline airflow obstruction or chronic respiratory symptoms†	
	Number of events	HR (95% CI) ‡	Number of events	HR (95% CI) ‡	Number of events	HR (95% CI) ‡	Number of events	HR (95% CI) ‡
**Major respiratory diseases§**								
Always clean	2,149	1.00 (0.95-1.05)	2,381	1.00 (0.96-1.05)	2,095	1.00 (0.95-1.05)	2,407	1.00 (0.96-1.05)
Solid to clean	4,280	1.15 (1.11-1.19)	4,107	1.14 (1.10-1.18)	4,050	1.12 (1.09-1.15)	4,176	1.15 (1.12-1.19)
Always solid	12,307	1.37 (1.32-1.41)	10,900	1.35 (1.30-1.40)	11,673	1.34 (1.29-1.39)	11,064	1.37 (1.32-1.42)
**Chronic lower respiratory diseaseǁ**								
Always clean	958	1.00 (0.93-1.07)	1,019	1.00 (0.94-1.07)	874	1.00 (0.93-1.08)	985	1.00 (0.93-1.07)
Solid to clean	2,119	1.19 (1.13-1.24)	2,041	1.19 (1.14-1.25)	2,011	1.18 (1.13-1.23)	2,035	1.25 (1.20-1.31)
Always solid	6,974	1.45 (1.40-1.51)	6,156	1.43 (1.38-1.49)	6,586	1.44 (1.37-1.51)	6,104	1.51 (1.45-1.58)
**Chronic obstructive pulmonary disease¶**								
Always clean	304	1.00 (0.89-1.13)	329	1.00 (0.89-1.13)	298	1.00 (0.88-1.14)	297	1.00 (0.88-1.13)
Solid to clean	738	0.97 (0.90-1.06)	674	0.94 (0.87-1.03)	697	0.94 (0.88-1.01)	630	1.00 (0.92-1.09)
Always solid	3,196	1.12 (1.04-1.20)	2,631	1.08 (1.00-1.17)	3,092	1.10 (1.01-1.19)	2,477	1.16 (1.07-1.26)
**Acute lower respiratory infection****								
Always clean	842	1.00 (0.93-1.08)	953	1.00 (0.93-1.08)	875	1.00 (0.92-1.08)	976	1.00 (0.93-1.07)
Solid to clean	1,762	1.10 (1.05-1.16)	1,640	1.07 (1.02-1.13)	1,667	1.06 (1.02-1.11)	1,707	1.07 (1.02-1.13)
Always solid	4,306	1.18 (1.11-1.25)	3,718	1.18 (1.11-1.26)	4,159	1.15 (1.07-1.24)	3,869	1.15 (1.08-1.23)
**Acute upper respiratory infection††**								
Always clean	364	1.00 (0.90-1.12)	419	1.00 (0.90-1.11)	347	1.00 (0.89-1.13)	429	1.00 (0.90-1.11)
Solid to clean	547	1.15 (1.06-1.26)	542	1.13 (1.04-1.24)	510	1.16 (1.06-1.26)	561	1.07 (1.04-1.24)
Always solid	1,929	1.63 (1.52-1.75)	1,766	1.52 (1.41-1.64)	1,727	1.64 (1.50-1.79)	1,856	1.56 (1.45-1.68)
**Other upper respiratory disease‡‡**								
Always clean	267	1.00 (0.88-1.14)	297	1.00 (0.88-1.13)	258	1.00 (0.87-1.15)	318	1.00 (0.88-1.13)
Solid to clean	388	1.08 (0.97-1.20)	394	1.15 (1.03-1.28)	367	1.12 (1.01-1.23)	404	1.09 (0.98-1.21)
Always solid	947	1.55 (1.39-1.73)	861	1.50 (1.33-1.68)	862	1.67 (1.46-1.91)	902	1.51 (1.35-1.69)
**Respiratory death§§**								
Always clean	49	1.00 (0.75-1.34)	46	1.00 (0.74-1.35)	40	1.00 (0.73-1.37)	39	1.00 (0.72-1.39)
Solid to clean	117	0.85 (0.68-1.06)	111	0.97 (0.78-1.22)	113	0.85 (0.68-1.06)	98	1.03 (0.80-1.32)
Always solid	446	1.46 (1.20-1.79)	332	1.35 (1.08-1.68)	426	1.39 (1.14-1.68)	332	1.99 (1.56-2.54)

* Weekly-regular cooks: participants reporting cooking weekly at baseline (n = 25,466)
† Airflow obstruction defined by spirometry; chronic respiratory symptoms: cough with phlegm
‡ Hazard ratios were stratified for age-at-risk, sex and study area and adjusted for education, household income, occupation, alcohol consumption, body-mass index, environmental tobacco smoke, cookstove ventilation, primary heating fuel, and length of recall period.
§ ICD-10 code J00-J06, J12-J18, J30-J22, J30-J39, J40-J47.
ǁ ICD-10 code J40-47.
¶ ICD-10 code J41-44.
** ICD-10 code J12-J18, J20-J22.
†† ICD-10 code J00-J06.
‡‡ ICD-10 code J30-J39.
§§ ICD-10 code J00-J47, J80-J94, J96-J99.

Figure E1. Locations of the 10 study areas of the China Kadoorie Biobank Study*

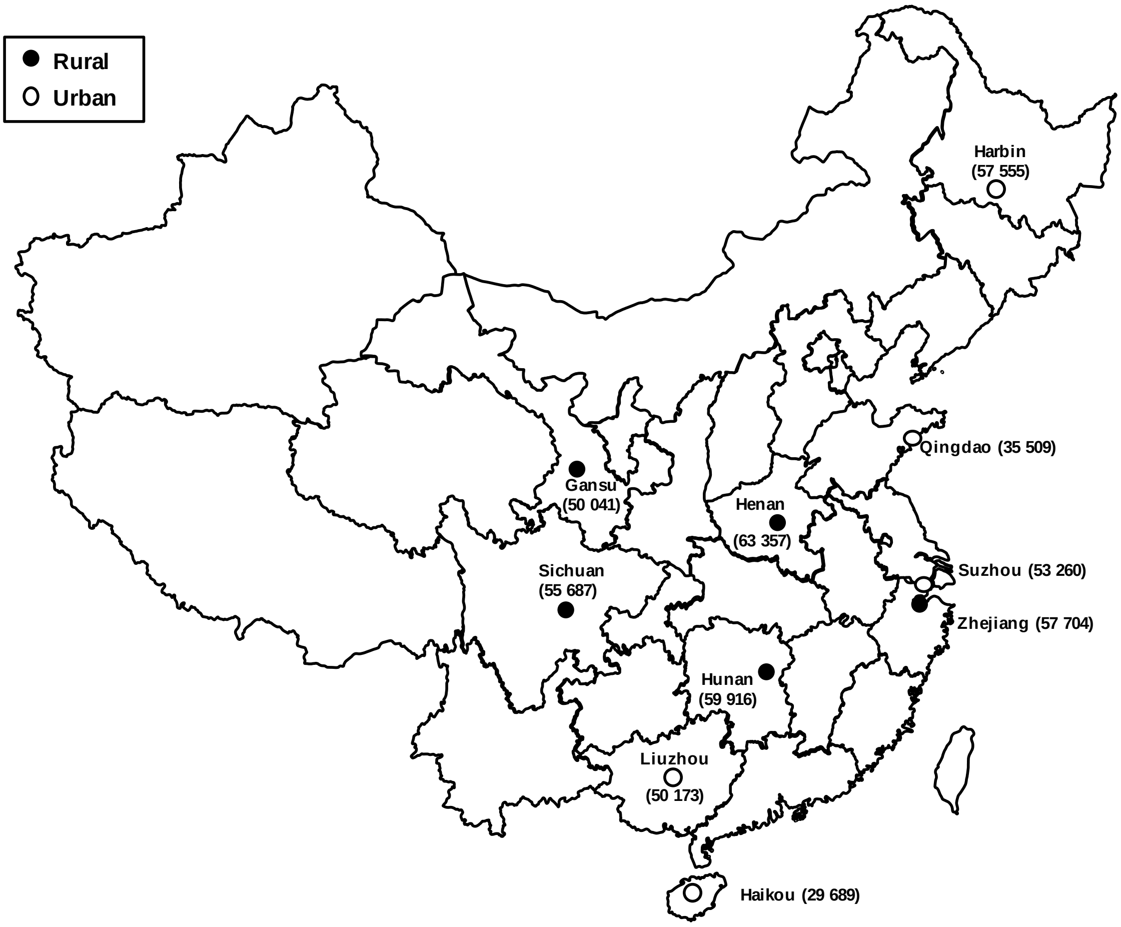

* Solid circles (●) are rural areas and open circles (○) are urban areas included in the China Kadoorie Biobank Study. Number of participant at baseline in each study area is shown in brackets.

References

1. Hong LS, Lewington S. Lexis Expansion- Age-at-risk adjustment for survival analysis. SAS Conference Proceedings: Pharmaceutical Users Software Exchange 2013. Available online: https://www.lexjansen.com/phuse/2013/sp/SP09.pdf.
2. Plummer M. Improved estimates of floating absolute risk. Stat Med. 2004;23(1):93-104.
3. Smith M, Li L, Augustyn M, Kurmi O, Chen J, Collins R, et al. Prevalence and correlates of airflow obstruction in approximately 317,000 never-smokers in China. Eur Respir J. 2014;44(1):66-77.
4. Kurmi OP, Davis KJ, Lam KB, Guo Y, Vaucher J, Bennett D, et al. Patterns and management of chronic obstructive pulmonary disease in urban and rural China: a community-based survey of 25 000 adults across 10 regions. BMJ Open Respir Res. 2018;5(1):e000267.
